# Children’s peer relationships, well-being, and academic achievement: the mediating role of academic competence

**DOI:** 10.3389/fpsyg.2023.1174127

**Published:** 2023-05-12

**Authors:** Ana-Maria Țepordei, Alexandra S. Zancu, Loredana R. Diaconu-Gherasim, Irina Crumpei-Tanasă, Cornelia Măirean, Dorina Sălăvăstru, Adrian V. Labăr

**Affiliations:** Faculty of Psychology and Educational Sciences, Alexandru Ioan Cuza University, Iași, Romania

**Keywords:** peer relationships, life satisfaction, achievement, academic competence, primary school

## Abstract

The present study aimed to explore the interplay among two indicators of children’s school peer relationships (i.e., peer acceptance and perceived number of friends) and two significant life domains (i.e., global life satisfaction and academic achievement). We also explored the potential mediating role of the perceived academic competence in these relations. Participants were 650 Romanian primary school students (45.7% boys), aged between 9 and 12 years old (*M*_age_ = 10.99). Path analysis showed a direct positive effect of perceived number of friends on children’s life satisfaction, as well as a direct positive effect of peer acceptance on academic achievement. Moreover, perceived academic competence mediated the links between each of the two indicators of peer relationships and children’s both life satisfaction and achievement. Several implications in the educational contexts are discussed.

## 1. Introduction

Theoretical models (e.g., bioecological framework; Bronfenbrenner, 1994) state that, besides their families, school is an important microsystem shaping children’s cognitive and socioemotional development. A proper school climate, defined as the quality of school life, can positively impact children’s cognitive and behavioral acquisitions, self-concepts and motivation, as well as their emotional well-being (e.g., Buhs, 2005; Flook et al., 2005; Boulton et al., 2011; Lester and Cross, 2015). Different domains of school climate have been distinguished in the literature, such as school facilities, safety, school connectedness, and social relationships (Zullig et al., 2011). The present study focuses on the latter domain, with a more specific interest in the impact of school peer relationships on primary school students’ academic and emotional outcomes. There is evidence that peer experiences are an important part of the school context for children and early adolescents, significantly contributing to their school liking, academic achievement, and wellness (e.g., Wentzel et al., 2004; Boulton et al., 2011; Oberle and Schonert-Reichl, 2013; Sebanc et al., 2016). Generally, the benefits of positive peer relationships on children’s both academic outcomes and well-being can be accounted for through their instrumental value (by offering support, access to peer activities, help in various tasks), or their affective value (when peers’ acceptance and support meet students’ psychological needs of affiliation and security; Ladd et al., 1997; Wentzel et al., 2021).

In this study, we addressed peer relationships at group level rather than on dyadic level, as we were more interested in exploring them as reflecting the general socio-emotional classroom climate seen as a more or less warm, comforting, and welcoming place where children spend a significant amount of their daily time, influencing their development (Boulton et al., 2011; Sebanc et al., 2016). Consequently, we chose to assess peer acceptance in terms of preference and not in terms of popularity, also considering that these two facets of peer status become more distinct during development and that popularity is of more concern in adolescence than in childhood (see van den Berg et al., 2020 for a meta-analysis). Following the same logic, we were interested in assessing “whether a child is liked” rather than “what a child is like” (Bukowski et al., 2012), meaning that we chose to measure peer acceptance by peer ratings and not by peer nominations. When using rating scores, each member of the group receives an equal number of evaluations from the other classmates, whereas nominations scores are limited to a smaller number of classmates, with the more salient children receiving many nominations and many other children receiving any nomination (Wentzel et al., 2021). Since peer ratings are external/objective evaluations from the others’ perspective, we used a second indicator grasping the child’s subjective perspective of the group socio-emotional climate, namely how many friends he or she thinks to have among classmates (i.e., unilateral number of friends). We reasoned that the more friends one feels to have in a group (even if not necessarily reciprocated), the more comforting and secure that group is perceived to be. Each of the two indicators of peer relationships was entered into further analyses as a separate variable, as previous research showed that different indicators of peer relationships might differently associate with various aspects of children’s school adjustment (Ladd et al., 1997).

Correlates of relations with peers were investigated more in samples of secondary and high school students, with research on primary school students especially addressing the role of social functioning and peer relationships as protective or risk factors over the transition to secondary school (e.g., Pellegrini and Bartini, 2000; Lester and Cross, 2015). Our study adds to the existing research by exploring, in a two-folded manner, the emotional and academic correlates of primary school students’ peer relationships. Specifically, we investigated how two indicators of relationships with peers - a subjective (i.e., reported number of friends in the class) and an objective indicator (i.e., peer acceptance based on peers’ ratings) - simultaneously predicted students’ life satisfaction and their academic achievement. Moreover, our study contributes to a better understanding of the underlying mechanisms of these links, by exploring the potential mediating role of perceived academic competence on the relations between peer relationships and life satisfaction and academic achievement.

### 1.1. Peer relationships and life satisfaction

The significance of positive peer relationships in promoting mental health and well-being among children and adolescents has been recognized for a long time in the literature (e.g., interpersonal theory; Sullivan, 1953), with more recent studies continuing to confirm it (see Schwartz-Mette et al., 2020 for a meta-analysis). Moreover, previous studies also highlighted the long-lasting effects of peer experiences across adolescence on subsequent long-term adjustment, mental health, and overall life satisfaction (e.g., Bagwell et al., 1998; Kekkonen et al., 2020). Existing findings showed that better-accepted children have a larger pool of peers who like them and better social skills to form lasting relationships (Bukowski et al., 1996). Through the size and also quality of their friendship networks, they gain greater protection from feelings of loneliness and isolation (e.g., Nangle et al., 2003). The perceived number of friends was found to be negatively associated with preadolescents’ depressive symptoms, and positively correlated with their happiness (e.g., Uusitalo-Malmivaara and Lehto, 2013). However, very few studies investigated the associations between children’s well-being and their number of friends assessed by unilateral nominations (see Schwartz-Mette et al., 2020 for a meta-analysis). Regarding peer acceptance, previous findings showed that higher levels of being or feeling accepted and liked by school peers were associated with higher levels of self-worth, as well as with lower levels of anxiety, loneliness and depression (e.g., Nangle et al., 2003; Bukowski et al., 2010). There is some evidence that peer acceptance is a positive predictor of children’s emotional adjustment (e.g., Hymel et al., 1990), most of previous studies focusing more on the negative indicators of mental health (i.e., internalizing problems).

Therefore, in the present study we simultaneously explored the role of peer sociometric assessment of acceptance and children’s self-reported number of friends on children’s overall life satisfaction. Thus, we highlight the significance of children’s being liked and feeling to have friends among school peers on a positive indicator of their subjective well-being that provides insight into their global happiness and perceived quality of life (Gilman and Huebner, 2003).

### 1.2. Peer relationships and academic achievement

Several theoretical models (e.g., social cognitive theory; Bandura, 1993; situated expectancy-value theory; Eccles and Wigfield, 2020; competence motivation theory; Harter, 1982), suggest that indicators of students’ social relationships within school need to be taken into account when studying academic achievement in early adolescence. Significant links between peer relationships and academic achievement are expected to be especially strong for primary school students, as their integration in the school group is of main concern during this developmental stage.

Previous studies found that being more liked in their school groups might positively impact early adolescents’ academic outcomes, especially through more support from their peers (see Wentzel et al., 2021 for a meta-analysis). For example, Gallardo et al. (2016) found peer acceptance (measured by free nominations) to positively predict subsequent academic achievement, the effect being stronger for early adolescents than for mid-adolescents. In another study, Kingery et al. (2011) showed that pretransition peer acceptance (measured by peer ratings) and number of reciprocated friends (measured by an unlimited nomination procedure) predicted the post-transition academic achievement, with the most robust links being between peer acceptance and early adolescents’ academic achievement. There are also studies indicating that peer acceptance (measured by number restricted nominations) did not predict students’ subsequent academic achievement during primary or middle school (e.g., Véronneau et al., 2010).

One aspect that might account for these contradictory results would be the different measures used for peer acceptance (sociometric assessment, free or number restricted peer nominations). Therefore, more studies are needed to explore how both subjective and objective indicators of the relations with peers are linked with students’ academic achievement. Given these mixed findings, an insightful way of clarifying these links would also be to go beyond the associations between peer relationships and academic achievement and to explore more the underlying mechanisms (i.e., mediators) that could account for these links.

### 1.3. The mediating role of perceived academic competence

Various individual factors might be influenced by peer dynamics having, in turn, a significant impact on students’ emotional well-being and academic achievement (Wentzel and Caldwell, 1997; Wentzel et al., 2021). Several studies explored such mechanisms in samples of children and early adolescents. For example, students’ self-concepts were found to mediate the impact of school-related social support from classmates, teachers, and parents on students’ life satisfaction and subjective well-being in school (e.g., Danielsen et al., 2009; Tian et al., 2015). Moreover, peer relationships were found to have an indirect impact on academic achievement mediated by various aspects, such as students’ behavioral and psychological engagement (e.g., Dotterer and Lowe, 2011), prosocial behavior (e.g., Wentzel and Caldwell, 1997), school belonging (e.g., Delgado et al., 2016), or self-beliefs (e.g., Buhs, 2005).

Adding to these findings, we argue that primary school student perceived academic competence might also explain the links between peer acceptance and self-reported number of friends with students’ both life satisfaction and academic achievement. As we are to illustrate below, there is quite consistent evidence for the associations between perceived academic competence and each of the aforementioned variables taken separately (except for the unilateral number of friends, an indicator very rarely used in previous research).

Theoretical models (e.g., social cognitive theory; Bandura, 1993; situated expectancy-value theory; Eccles and Wigfield, 2020; competence motivation theory; Harter, 1982) emphasize that peer relationships might significantly impact children’s self-evaluative beliefs in the academic domain, apart from the influences of their parents and teachers. Previous research indicated that both peer acceptance and number of friends (i.e., reciprocated) were positively correlated with children’s self-esteem before and after transition to middle school (Kingery et al., 2011). Further, positive associations were consistently found between peer acceptance and perceived academic competence, whereas peer rejection had a negative impact on children’s academic self-concept (see Wentzel et al., 2021 for a meta-analysis). Importantly, children’s perceptions of their academic competence were significant positive predictors of their overall life satisfaction (e.g., Leung et al., 2004). The greater the confidence in one’s abilities to successfully manage academic work, the higher the positive feelings toward oneself, happiness and overall satisfaction with one’s life (e.g., Huebner et al., 1999; Chang et al., 2003). Studies also showed that academic self-concept is more strongly related to children’s subjective well-being than other objective indicators or external ratings of academic competence (e.g., Huebner and Alderman, 1993).

Regarding the relation between perceived academic competence and academic achievement, there is a strong, well-documented agreement among researchers, who consistently reaffirm the positive association between these constructs (e.g., Harter et al., 1992; Bandura, 1993; Eccles and Wigfield, 2020). Students’ academic self-concept was found to play a substantial role in their academic success, beyond the influence of prior achievement, test scores, or other indicators of cognitive abilities (e.g., Bouffard-Bouchard, 1990 Bandura et al., 2001 Marsh and Hau, 2003). In other words, a student with a positive mindset, confident in his or her ability to do well and manage academic tasks will be more motivated and persistent, thus enhancing the probability of higher levels of academic achievement. Notably, this positive impact of perceived academic competence on academic achievement was consistently found across various developmental stages and normative transitions in the school system (see Wentzel et al., 2021 for a meta-analysis). For example, Roeser et al. (1999) found that negative perceptions of academic competence had a significant impact on the continuity of problematic patterns of students’ achievement and mental health throughout the school years.

Nonetheless, despite of the findings synthetically presented above, there is a surprising paucity of studies exploring whether the relations of primary school students’ peer acceptance with both life satisfaction and achievement is mediated by their perceived academic competence. Moreover, to our knowledge, there is no previous study addressing whether the perceived competence may play a mediating role on the relation between number of friends and children’s adjustment.

Wentzel et al.’ s (2021) comprehensive metanalysis showed that students’ academic self-beliefs partially mediated the relation between peer acceptance and their academic achievement. However, it is important to emphasize here that out of the 72 studies which were entered in the final analysis sample, only eight simultaneously considered peer acceptance, academic competence, and achievement. Furthermore, only four of them addressed primary school students, and only one used peer ratings (and not peer nomination) for assessing school peer acceptance. This latter study (Buhs, 2005) showed that peer acceptance negatively predicted both peer victimization and peer exclusion, that further negatively predicted academic self-competence, which in turn partially mediated the relations of the forms of negative peer treatment with academic achievement. In addition, another study showed that peer rejection assessed by teachers in fourth grade predicted lower levels of academic self-concept which, further predicted lower academic achievement in 6^th^ grade (Flook et al., 2005). Although these studies addressed the more aversive or problematic forms of peer relationships (i.e., victimization, exclusion, rejection), they support the mediational role of children’s self-related beliefs for the link between peer relationships and academic achievement.

### 1.4. The present study

Two main objectives guided the present research. First, we explored more deeply the interplay between two indicators of school peer relationships (i.e., peer acceptance and self-reported number of friends) and two significant children’s adjustment indicators (i.e., life satisfaction and academic achievement) in a sample of Romanian 4^th^ grade primary school students. Second, we investigated whether perceived academic competence might play a significant mediating role in these associations.

Our study focuses on a preadolescent group for several reasons. First, there are very few studies addressing simultaneously these specific aspects related to primary school students’ academic adjustment and subjective well-being. Second, we wanted to enrich our understanding of the children’s functioning through a transversal “radiography” in their present school peer groups *per se*, and not necessarily in the relation with their upcoming transition to middle school and its associated changes in the academic settings. Third, at this age group level, relationships with peers were found to have a significantly stronger association with academic achievement (Wentzel et al., 2021), and the latter, together with academic self-concept, to be strongly related with peer acceptance and life satisfaction (e.g., Chang et al., 2003; Leung et al., 2004; Buhs, 2005). Fourth, in the Romanian educational context, primary school comprises of 5 years (from preparatory class to 4th grade), with the classroom structure remaining almost the same from the beginning and with the same teachers in most of the cases. Therefore, the school peer groups in our sample have a highly stable composition, with classmates knowing each other very well, most of them having been together since the preparatory class. This means an almost 5-years period of daily interactions, common academic tasks and extra-curricular activities, lunch breaks, school playgrounds etc., rendering peer relationships very important for children’s both academic and well-being.

Therefore, our study aims at contributing to the existing literature by investigating these aspects in an underrepresented sample (i.e., Romanian primary school children) and by addressing less explored indicators of school peer relationships (i.e., unilateral number of friends and peer acceptance). Moreover, it contributes to the field by investigating the mechanisms (i.e., academic self-concept) that might explain the impact of peer experiences on children’s adjustment (i.e., life satisfaction and academic achievement). To conclude, we hypothesized the followings: 1. A higher level of peer acceptance and a larger number of self-reported friends, respectively, would be associated with both a higher level of life satisfaction and a better academic achievement; 2. The perceived academic competence would mediate the links of both peer acceptance and number of friends with life satisfaction and achievement, in the sense that higher levels of peer acceptance and a larger number of self-reported friends would be related to higher levels of perceived academic competence which, in turn, would lead to higher levels of both overall life satisfaction and academic achievement.

## 2. Materials and methods

### 2.1. Participants

The sample included 650 fourth grade children (45.7% boys), aged between 9 and 12 years old (*M*_age_ = 10.99, SD = 0.35), recruited from seven public schools in a large city in Romania, where the study was conducted with the approval and collaboration of the local authorities. The school size ranged between 3 to 7 classes with 85 to 200 students per each grade level (with an average of 30 students per class). The schools were similar in terms of school organization, school performance ratings, and counseling programs. For 87.1% of the children, the parents reported intact family status, while 12.9% were single parents (divorced/separated, or widowed). Regarding parental education, 4.3% of the mothers and 10.9% of the fathers have less than a high school degree, 22.6% of the mothers and 24.8% of the fathers have a high school degree, while 73.1% of the mothers and 64.3% of the fathers have a university degree. Mothers’ and fathers’ education levels were positively associated (*r* = 0.62*, p <* 0.001), thus were averaged into an overall parental education level. Regarding parental income, 9.3% of the mothers and 5.8% of fathers have low-level income (less than 300 Euros per month), 36.8% and 33.1% have a medium-level income (between 300 and 700 Euros per month), while 53.9% of mothers and 61.1% of fathers have high-level income (more than 700 Euros per month). Parents’ income levels were significantly related (*r =* 0.44, *p* < 0.001) and were combined into an overall parental income level.

### 2.2. Procedure

The protocol for this study was approved by the Institutional Research Ethics Committee of the university where the authors are affiliated. The director of each participating school granted permission for conducting the study. Then, informative invitation letters were sent to the parents of all 4^th^ grade students in these schools. Those who agreed with the participation of their children signed the informed consent. The acceptance rate was 74.71%. Children also signed an informed consent before completing the questionnaires assessing peer acceptance, self-reported number of friends, perceived academic competence, and life satisfaction. Children’s grades were collected at the end of the semester from the school registers. Both parents and children were informed about the confidentiality of their answers and that they could withdraw from the study at any moment. Students completed the measures in their classrooms, alongside with their classmates, during their regular school hours, in the last 3 months of the 4^th^ grade. A previously trained school counselor presented the instructions and assisted the children in completing the measures. For their participation, children received school supplies.

### 2.3. Measures

For this study, two bilingual researchers independently used the forward-backward method to translate the questionnaires from English into Romanian, with differences in translations being discussed among the team members until consensus was achieved (Sousa and Rojjanasrirat, 2011). Each scale was reviewed for accuracy, semantics, cultural relevance, and age appropriateness. Next, we had the final questionnaires checked by 10 fourth-grade children who reported any ambiguous or age-inappropriate constructions. Based on their input, we further adapted the final version of the measures.

#### 2.3.1. Unilateral number of friends

Similar to other studies (e.g., George and Hartmann, 1996), children were asked to answer two sociometric items: (1) “*How many friends do you have in your class?*” and (2) “*Please name these friends*.” Children could nominate any classmate, regardless of his/ her participation in the study. Each participant’s self-reported number of friends was entered as a measure for this variable. Previous research found that unilateral friendship nominations had similar associations with friendship quality and mental health as reciprocal nominations (Schwartz-Mette et al., 2020).

#### 2.3.2. Peer acceptance

Peer acceptance was assessed using the peers’ ratings procedure (Bukowski et al., 2012). Children were provided with a roster listing the names of their classmates (i.e., only of those with parental consent to participate) and were asked to respond to the question, “*How much do you like each of these classmates?*.” Children were instructed to rate each of their peers from the list on a 5-point Likert scale ranging from 1 (not at all) to 5 (very much). For each student, a peer acceptance score was computed by averaging all the ratings received from their classmates participating in the study. The number of participating students ranged between 10 and 30 (*M* = 18.02, SD = 5.73) across classes, the average classroom size being of 30 students. Unlike most of the previous studies using peer ratings where students were asked to evaluate how much they like *to play with/ work with during academic activities* each of their classmates, we chose not to direct children’s answers on a specific criterion (e.g., Boulton et al., 2011).

#### 2.3.3. Life satisfaction

Children’s overall life satisfaction was assessed using the Brief Multidimensional Students’ Life Satisfaction Scale (BMSLSS; Seligson et al., 2003), a 5-item self-report measure assessing different domains of life satisfaction, including academic, family, peer, self, and living environment (e.g., *I would describe my satisfaction with my family life as*). Items are rated on a 7-point Likert scale, ranging from 1 (terrible) to 7 (delighted). All items are then averaged into an overall score, with higher scores indicating higher levels of overall life satisfaction (*α* = 0.77). Previous studies indicated high levels of reliability and validity of the scale (e.g., Seligson et al., 2003; Huebner et al., 2006).

#### 2.3.4. Perceived academic competence

The 6-item Scholastic Competence subscale of the Self-Perception Profile for Children (Harter, 1982) was used for assessing students’ perceptions on their academic competence. Each item consists of two opposite descriptions of children with high vs. low academic competence (e.g., *Some kids feel they are very good at their school work BUT Other kids worry about whether they can do the school work assigned to them*) and children are asked to select which option fits them best and then indicate whether the statement is *sort of true* or *true* for them. The items are then scored on a 4-point scale ranging from 1 to 4 and averaged into an overall score, with higher scores indicating higher perceived academic competence (α = 0.79). Previous studies confirmed the reliability of the scale and exhibited good convergent validity with measures of the school environment (e.g., Harter, 2012; Ferro and Tang, 2017).

#### 2.3.5. Academic achievement

Students’ academic achievement was assessed by averaging their final scores in Math and Romanian language at the end of the year, collected from the school registers. In primary school, four qualitative evaluations are used, ranging from 1 (insufficient) to 4 (very good).

### 2.4. Overview of statistical analyses

Using IBM SPSS 26, we first conducted a missing value analysis (Little and Rubin, 2019), as well as preliminary analyses to examine the relations between demographic data and main study variables. Because participants were nested within schools, we used intraclass correlations analysis (ICC) to test for between-school variations in children’s life satisfaction scores and academic achievement (Peugh, 2010). Further, descriptive statistics and zero-order correlations among the main study variables were computed. Skewness and kurtosis were computed to assess normality of distribution for the study variables, based on the recommended values ranges for normal distribution between −2 and + 2 for skewness and − 10 to +10 for kurtosis (Collier, 2020). To test the study hypotheses, we conducted a path analysis using IBM AMOS Graphics 20 to simultaneously examine the main effects of peer acceptance and self-reported number of friends on children’s life satisfaction and academic achievement, as well as the mediating role of perceived academic competence in these relations.

The demographic variables significantly related with outcome variables in preliminary analyses were used as covariates in the model. We used full information maximum likelihood (FIML) estimations to account for missing data (Enders, 2010). The overall model fit was assessed using chi-square statistics (*χ*^2^) and three model fit indexes: the normed fit index (NFI), the comparative fit index (CFI), and the root mean square error of approximation (RMSEA). A *χ*^2^/df < 3, NFI and CFI values >0.95, and a RMSEA <0.05, respectively, indicate a very good model fit (Hu and Bentler, 1999). To examine the significance of the mediation effects, we computed the 95% confidence intervals for the indirect effects based on Tofighi and MacKinnon’s (2011) method. The indirect effect is significant when the confidence intervals do not include zero (Collier, 2020).

## 3. Results

### 3.1. Preliminary analyses

Missing data analysis indicated that data were missing at random, with no systematic difference for any of the main study or demographic variables, *χ*^2^ (156) = 156.38, *p* = 0.47. Intraclass correlations indicated no significant between-school variability in either life satisfaction scores (ICC = 0.08, *p =* 0.465), or academic achievement (ICC = 0.01, *p =* 0.14), suggesting that data from all schools could be combined for main analyses. Skewness and kurtosis values for all the study variables were in the recommended acceptable range for normal distribution (see Table 1).

**Table 1:** Descriptive statistics and zero-order correlations among the main variables.

Variables	*N*	*M* (SD)	Min—Max	Skew	Kurt	1	2	3	4
1. Peer acceptance	639	3.34 (0.54)	1.17–6.00	−0.23	1.31				
2. Unilateral number of friends	640	8.75 (6.84)	0–38	1.77	3.47	0.15***			
3. Academic competence	643	2.89 (0.68)	1.00–4.00	−0.32	−0.29	0.14***	0.17***		
4. Life satisfaction	627	28.53 (5.08)	5.00–35.00	−0.98	1.02	0.07	0.18***	0.40***	
5. Academic achievement	623	3.70 (0.51)	2.00–4.00	−1.68	1.97	0.34***	0.11**	0.28***	0.12**

*p < 0.05, **p < 0.01, ***p < 0.001.

Demographic analyses indicated significant gender difference in academic achievement, *t* (621) = −3.18, *p = 0*.002, girls having higher grades, *M* (SD) = 3.76 (0.47) than boys, *M* (SD) = 3.62 (0.55). However, girls reported lower levels of perceived academic competence, *M* (SD) = 2.82 (0.70) than boys, *M* (SD) = 2.97 (0.64), *t* (641) = 2.72, *p =* 0.007. No significant gender differences were found in life satisfaction, *t* (625) = 0.24, *p =* 0.81. Children’s age was not significantly related with any of the main study variables (*r*s < 0.08, all *p* > 0.05). Significant differences were found in life satisfaction scores based on family status, *t* (601) = 2.35, *p = 0*.02, with children from intact families having higher scores, *M* (SD) = 28.66(4.87), than children from single parent families, *M* (SD) = 26.94 (6.12). Similarly, significant differences were found in academic achievement based on family status, *t* (598) = 2.98, *p =* 0.004, with children from intact families having higher grades, *M* (SD) = 3.73 (0.48) than children from single parent families, *M* (SD) = 3.51 (0.63). Parental education was positively related with children’s academic achievement (*r* = 0.45, *p <* 0.001) and perceived academic competence (*r* = 0.14, *p* = 0.001), but not with children’s life satisfaction (*r* = 0.08, *p* > 0.05). Parental income level was also positively associated only with children’s academic achievement (*r* = 0.32, *p* < 0.001), but not with their life satisfaction (*r* = 0.05, *p* = 0.25).

### 3.2. Associations among the main study variables

Descriptive statistics and zero-order correlations among the main study variables are presented in Table 1. Peer acceptance was positively associated with academic achievement but not significantly associated with life satisfaction. The unilateral number of friends was positively correlated with life satisfaction and academic achievement. Both peer acceptance and the unilateral number of friends were positively associated with perceived academic competence. Further, perceived academic competence was positively associated with both life satisfaction and academic achievement (see Table 1).

### 3.3. Path analysis

Path analysis simultaneously tested the main effects of peer acceptance and unilateral number of friends on children’s life satisfaction and academic achievement, as well as the mediating role of perceived academic competence. Demographic variables (i.e., gender, family status, parental education, and parental income) were introduced as covariates in the model, due to their significant relations with the outcome variables. Results of the path analysis for the hypothesized model indicate a very good fit to the data: *χ*^2^(15) = 16.67, *p* = 0.33, *χ*^2^/df = 1.11; NFI = 0.97; CFI = 0.99, RMSEA = 0.01, [CI: 0.00; 0.04]. The model explains 17.6% of the variance in children’s life satisfaction scores, 32.3% of the variance in academic achievement, and 6.6% of the variance in perceived academic competence.

**Figure 1:**
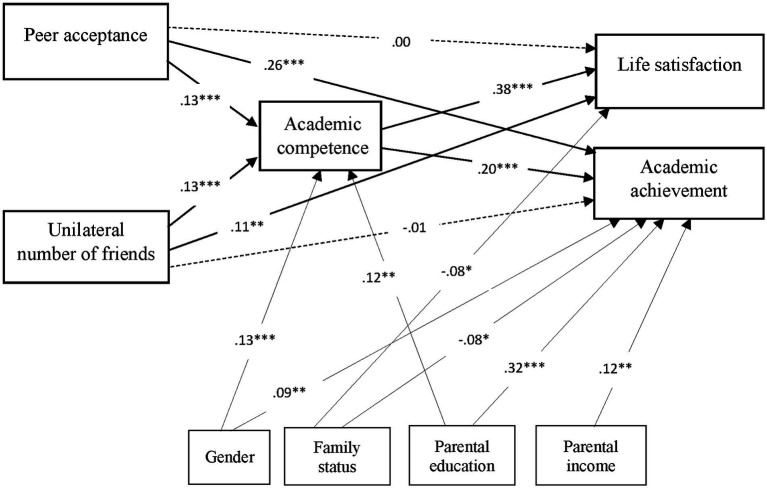
Path analysis testing the study hypotheses (*N* = 650). Standardized path coefficients are reported. *<0.05, **<0.01, ***<0.001.

As depicted in Figure 1, both peer acceptance (*β* = 0.13, *p* < 0.001) and unilateral number of friends (*β* = 0.13, *p* < 0.001) were significantly positively related with perceived academic competence, which was further positively related with both life satisfaction (*β* = 0.38, *p* < 0.001) and academic achievement (*β* = 0.20, *p* < 0.001). In addition, peer acceptance was positively related with academic achievement (*β* = 0.26, *p* < 0.001), but was not significantly related with life satisfaction. By contrast, the unilateral number of friends was positively related with life satisfaction (*β* = 0.11, *p* = 0.005), but not with academic achievement. Gender, parental education and parental income were positively related with perceived academic competence and academic achievement.

Perceived academic competence significantly mediated the effects of both peer acceptance, indirect effect estimate = 0.464 (SE = 0.148), 95% CI [0.184, 0.767], and of unilateral number of friends, indirect effect estimate = 0.037 (SE = 0.012), 95% CI [0.014, 0.061], on children’s life satisfaction scores. It also mediated the effects of both peer acceptance, indirect effect estimate = 0.024 (SE = 0.009), 95% CI [0.009, 0.043], and of unilateral number of friends, indirect effect estimate = 0.002 (SE = 0.001), 95% CI [0.001, 0.003], on children’s academic achievement.

## 4. Discussion

The present study adds to the existing findings by simultaneously investigating the role of two important indicators of children’s relationships with school peers (i.e., peer acceptance and unilateral number of friends) on two key indicators of children’s adjustment (i.e., overall life satisfaction and academic achievement). We also advance the literature by exploring the potential mediating role of students’ academic self-concept in these relations.

In line with our hypothesis, correlational and path analyses indicated that children who report having more friends among their classmates are generally more satisfied with their lives. This result is convergent with previous research showing that children’s friendship network size (i.e., number of friends), either reciprocated or just subjectively perceived (i.e., unilateral), can have beneficial consequences on their well-being (see Schwartz-Mette et al., 2020 for a meta-analysis). Our findings confirm the idea that those children who perceive having more friends within their school groups, with whom they share a significant amount of time every day, are more likely to have positive interactions with their peers (Bukowski et al., 1996) and to have their psychological needs for affiliation and security met, thus feeling less isolated, lonely or depressed (Nangle et al., 2003). Consequently, prevention and intervention school-based programs (e.g., contact interventions; Echols and Ivanich, 2021) could be implemented for developing and strengthening interpersonal openness and closeness among school peers, in order to prevent children from feeling lonely or socially isolated, and thus to increase their subjective well-being.

Regarding primary school students’ peer acceptance, results in our study did not support the expected link with overall life satisfaction. This finding might strengthen the idea that in preadolescence, the impact of peer acceptance, assessed by external peer ratings, on subjective well-being is weaker than the impact of the children’s perceived peer acceptance (e.g., Hymel et al., 1990; Nelson et al., 2005). This result might also be in line with the idea that preadolescents start to give more attention to their friendship relations, given that preadolescence is marked by an emergence of close friends that better satisfy children’s growing need of intimacy (Sullivan, 1953; van den Berg et al., 2020). As a result, children’s life satisfaction appears to be more affected by their perceived number of friends than by their classmates’ degrees of personal preference.

Concordant with previous research (e.g., Oberle and Schonert-Reichl, 2013; Gallardo et al., 2016), correlational analysis indicated that both higher levels of peer acceptance and a greater self-reported number of friends were related with better academic achievement. Path analysis, however, indicated that only peer acceptance was a significant predictor of children’s academic achievement. These findings confirm previous literature showing that peer acceptance is a strong positive predictor of children’s academic outcomes during primary school (see Wentzel et al., 2021 for a meta-analysis). They also reinforce the idea that, at this age, children show a higher concern regarding their integration in the school group, rendering group status to strongly impact their academic outcomes (Wentzel et al., 2004; Véronneau et al., 2010). Further, the unilateral number of friends was not a significant predictor of primary school students’ academic achievement. This result confirms some previous studies also showing that friend nomination is not directly related with academic achievement, and that these constructs are rather indirectly linked through other contextual factors, as school belonging (e.g., Delgado et al., 2016).

Nevertheless, taken all together, the results so far reveal some interesting nuances. Unilateral number of friends had a direct effect on children’s overall life satisfaction, but not on their academic achievement. Conversely, peer acceptance had a direct effect on children’s achievement, but not on their life satisfaction. These findings strengthen the previously suggested idea that different indicators of school peer relationships might differently impact various aspects of children’s school and emotional adjustment (Ladd et al., 1997). Moreover, these differentiated direct effects might also emphasize the fact that the benefits of positive peer relationships could be analyzed through their either instrumental or affective value (Ladd et al., 1997; Wentzel et al., 2021). Our findings suggest that self-reported number of friends might have a more affective value (meeting students’ psychological needs of belonging and cohesion) influencing more their subjective well-being, whereas peer acceptance might have a more instrumental value (more openness from colleagues, support, access to and help in various academic activities) influencing more their academic outcomes.

Despite growing evidence that school peer acceptance and number of friends are positively related to life satisfaction and academic achievement in pre-adolescence, the underlying mechanisms explaining these associations are less investigated (Delgado et al., 2016 Wentzel et al., 2021). To contribute to the existing literature, in the present study we investigated whether perceived academic competence might have a significant mediating role on the relations among these constructs. First, in line with previous research showing various indicators of peer relationships to be relevant predictors of children’s beliefs related to their competence in school (e.g., Altermatt and Pomerantz, 2003; Buhs, 2005), our results indicated that better-accepted children or those who reported having more friends among peers, also reported higher levels of perceived academic competence. Our findings advance previous literature by simultaneously investigating the impact of the two indicators of relationships with peers on academic self-concept. Moreover, children reporting higher levels of perceived academic competence declared themselves more satisfied with their lives and had higher levels of academic achievement. These results align with both theoretical models (social cognitive theory; Bandura, 1993; situated expectancy-value theory; Eccles and Wigfield, 2020; competence motivation theory; Harter, 1982) and previous empirical studies (e.g., Harter et al., 1992; Roeser et al., 1999; Chang et al., 2003; Leung et al., 2004) suggesting that students who are more self-confident in their abilities to manage the school work increase their chances for more successful academic activities, thus experiencing higher levels of self-worth, life satisfaction, and academic achievement.

An important result of our study is that perceived academic competence mediated the relations between both peer acceptance and number of friends with children’s overall life satisfaction. Specifically, both indicators of relationships with peers positively predicted children’s perceptions of their academic competence, that further positively predicted their life satisfaction. These results expand the existing knowledge by providing evidence for a psychological mechanism that links these two particular aspects of school peer experiences with children’s subjective well-being, adding to other previous finding indicating that school-related social support has a mediated impact on preadolescents’ life satisfaction through their self-concepts (e.g., Danielsen et al., 2009; Tian et al., 2015). Moreover, our study further contributes to the previous literature by showing that children’s perceived number of friends (regardless of their reciprocity) also might impact their academic self-concept and, further, their overall life satisfaction.

Further, perceived academic competence also mediated the relations between each of the two indicators of relationships with peers with academic achievement. Specifically, a greater number of self-reported friends or a higher level of peer acceptance positively predicted children’s perceptions on their academic competence, which further positively predicted their academic performance. These results confirm previous studies (see Wentzel et al., 2021 for meta-analysis) indicating that the association between students’ peer acceptance and academic achievement could be explained by their confidence in their abilities to successfully solve academic-related tasks. Further, our study also complements previous literature (e.g., Delgado et al., 2016) suggesting that the number of friends could be rather indirectly related to children’s academic achievement, throughout their academic self-beliefs.

However, several limitations should be considered. First, given the cross-sectional design of the study, our results cannot sustain causal relations between variables. Future longitudinal studies are needed in order to address the possible bidirectional effect between perceived academic competence and academic achievement (e.g., Bandura, 1993), or between peer acceptance and academic achievement (e.g., Véronneau et al., 2010). Second, children in our sample were recruited from a single urban area. Therefore, the external validity of the results is limited. In addition, given that many of these children live in intact families (with both parents), and that the majority of parents have higher levels of education and a high-level income it is important to be cautious when trying to generalize our findings to the broader Romanian primary school children population. It is generally known that these socio-demographic factors are important in explaining children’s life satisfaction and academic achievement (e.g., Gilman and Huebner, 2003; Eccles and Wigfield, 2020). Third, we globally assessed both perceived academic competence and academic achievement. This approach was useful in offering us a general picture about the interplay between school peer relationships, academic self-concept, and achievement, but for a deeper understanding of these relations, future studies could focus on different domains of academic self-efficacy and academic performance. Finally, we were focused on peer relationships at the group level, as an indicator of the socio-emotional classroom climate. However, we included in the study only those children whose parents gave their consent for participation. Therefore, the ratings for peer acceptance assessment could be limited by the fact that they did not reflect peer acceptance at the entire classroom level. Despite this limitation, we consider that including these external ratings is a strength of the study and the measurement was made according to the ethical standards about informed consent and based on the guidelines from the literature about social network evaluation (Bukowski et al., 2012). Moreover, we somehow counterbalanced this limitation by including the second indicator of school peer relationships, namely the self-reported number of friends among classmates, where unilateral nominations were unrestricted, as we were not interested in explorations at a dyadic level (reciprocated friendships or best friends’ nominations).

The overall results of the present study may have several important implications for educational contexts. First, they strengthen the idea that school settings are among the important microsystems significantly influencing children’s development, with its social, cognitive, and emotional aspects being highly interrelated (Bronfenbrenner, 1994). Although school is intuitively seen as being primarily focused on knowledge acquisition and cognitive skills, its role is (or should be) much larger than that. Educators and school counselors must acknowledge the fact that, for students, the cognitive process of learning actually takes place on a socio-emotional background that, depending on its quality, might either promote or hinder academic cognitive outcomes. As emphasized also by our findings, school peer dynamics may play an important role in providing the psychological conditions (e.g., affiliation, acceptance, support) that enhance school liking and learning (Ladd et al., 1997; Boulton et al., 2011). Therefore, school-based intervention strategies should also focus on improving school peer relationships, which might have a positive impact on students’ self-beliefs and self-confidence in their academic abilities, thus increasing not only their achievements, but also their overall life satisfaction. For example, peer-mediated behavioral interventions (Odom and Strain, 1984), or contact interventions (Echols and Ivanich, 2021) could be used for teaching children social and emotional skills, as a baseline for developing positive interactions with their classmates. The development of strong and protective school peer relationships system can also contribute to the prevention of school bullying by strengthening those aspects of children interactions that defend against internal or external sources of stress (Song and Stoiber, 2008). As previously shown, protective and supportive peer relationships could function as a safety net, effectively buffering against peer victimization (see Hong and Espelage, 2012 for a review).

Teachers could also use a wide range of in-class or extra-curricular activities targeting students’ supportive social relationships. On the one hand, such activities should leverage the affective value of peer relationships by giving students’ the opportunity to get to know each other better, to discover and acknowledge the others’ qualities, and to reinforce their own abilities and strengths. In this way, students’ positive aspects and common interests would be highlighted, thus increasing both peer acceptance levels and their number of friends. Enhanced cohesiveness and valorization among group members would also increase one’s self-worth and academic self-concept. On the other hand, peer-assisted cooperative learning activities should leverage the instrumental value of peer relationships by teaching students to accept one another, to communicate, and to provide support when needed (see Ginsburg-Block et al., 2006, for a meta-analysis). In this way, children might be more self-confident in their successfully managing a task, might fear failure less, also feeling less anxious or helpless. Altogether, this sort of activities could promote better school peer experiences and more positive perceptions on academic competence, which, in turn, might have a positive impact on children’s performance and subjective well-being.

As a conclusion, the present study highlights the positive relations between peer acceptance and academic achievement, and also the positive relations between self-reported number of friends and life satisfaction. Moreover, the results offer support for the mediating role of children’s perceived academic competence in the relation between each of the two indicators of school peer relationships with each of the two important indicators of children’s adjustment (i.e., life satisfaction and academic achievement). Strengthening positive school peer interactions could be a way of enhancing students’ self-confidence in their academic competence, consequently increasing both their subjective well-being and academic achievement. Future longitudinal research should add more evidence for the predictive and mediating role of academic self-concept in the relation of children’s interpersonal relationships with their affective and cognitive outcomes. Our findings should be taken one step further and enriched by future studies also considering other dimensions of children’s self-concept and other psychological aspects that might mediate or moderate these interconnections between school peer dynamics and students’ academic and emotional adjustment.

## Data availability statement

The raw data supporting the conclusions of this article will be made available by the authors, without undue reservation.

## Ethics statement

The studies involving human participants were reviewed and approved by Research Ethics Committee of the Faculty of Psychology and Educational Sciences, Alexandru Ioan Cuza University, Iasi, Romania. Written informed consent to participate in this study was provided by the participants’ legal guardian/next of kin.

## Author contributions

A-MȚ: conceptualization, methodology, writing—original draft, writing—review and editing, and writing—final manuscript. AZ: methodology, formal analysis, data curation, writing—original draft, and data collection. LD-G: conceptualization, methodology, writing—original draft, writing—review and editing, supervision, and funding acquisition. IC-T: writing—original draft, writing—review and editing, and data collection. CM: writing—original draft and data collection. DS: writing—original draft. AL: writing—original draft. All authors contributed to the article and approved the submitted version.

## Funding

The article processing fee was supported by authors’ institutional publication grants of the Faculty of Psychology and Educational Sciences, University “Alexandru Ioan Cuza” Iasi, Romania.

## Conflict of interest

The authors declare that the research was conducted in the absence of any commercial or financial relationships that could be construed as a potential conflict of interest.

## Publisher’s note

All claims expressed in this article are solely those of the authors and do not necessarily represent those of their affiliated organizations, or those of the publisher, the editors and the reviewers. Any product that may be evaluated in this article, or claim that may be made by its manufacturer, is not guaranteed or endorsed by the publisher.
